# Supplementary material for: Chemical Characterization and Quality Assessment of Copaiba Oil-Resin Using GC/MS and SFC/MS
Source: Plants (Basel). 2023 Apr 11;12(8):1619. doi: 10.3390/plants12081619 (PMC10144763; doi:10.3390/plants12081619)
Supplement: Supplementary file 1 [file plants-12-01619-s001.zip › plants-2311169-supplementary.pdf]

## Supplementary Materials

# Chemical Characterization and Quality Assessment of Copaiba Oil-Resin Using GC/MS and SFC/MS

Joseph Lee[1], Mei Wang[2*], Jianping Zhao[1], Zulfiqar Ali[1], Mohammed F. Hawwal[3], Ikhlas A. Khan[1,4*]

[1]National Center for Natural Products Research, School of Pharmacy, University of Mississippi, University, MS, 38677, USA

[2]Natural Products Utilization Research Unit, Agricultural Research Service, U.S. Department of Agriculture, University, MS, 38677, USA

[3]Department of Pharmacognosy, College of Pharmacy, King Saud University, Riyadh 4545, Saudi Arabia

[4]Division of Pharmacognosy, Department of BioMolecular Sciences, School of Pharmacy, University of Mississippi, University, MS, 38677, USA

**Table S1** Tentative compound identification and concentration (peak area %) of Copaiba oil-resin.

| No. | Compound* | $RRI^{Cal}$ | $RRI^{Lit}$ | 613 | 614 | 615 | 616 | 860 | 327 | 617 | 618 | 621 | 622 | 623 | 624 | 625 | 626 |
|---|---|---|---|---|---|---|---|---|---|---|---|---|---|---|---|---|---|
| | | | | *Authentic Samples* | | | | | *Unknown Samples* | | | | | | | | |
| 1 | δ-EIemene | 1342 | 1338 | 1.92 | 1.00 | 0.84 | 0.99 | 0.99 | 0.36 | 2.25 | 0.90 | 0.20 | 1.02 | 0.83 | 1.29 | 1.59 | 2.10 |
| 2 | α-Cubebene | 1355 | 1351 | 1.45 | 0.69 | 0.65 | 0.70 | 0.68 | 0.50 | 1.91 | 0.13 | - | 0.71 | 0.05 | 0.74 | 1.19 | 1.44 |
| 3 | α-Ylangene | 1379 | 1375 | 0.25 | 0.19 | 0.13 | 0.19 | 0.16 | 0.09 | 0.27 | 0.42 | 0.15 | 0.13 | 0.51 | 0.63 | 0.24 | 0.25 |
| 4 | α-Copaene | 1384 | 1376 | 11.25 | 9.27 | 7.33 | 9.29 | 9.24 | 5.36 | 11.25 | 9.57 | 3.66 | 7.84 | 7.14 | 10.11 | 11.96 | 10.52 |
| 5 | 7-epi-Sesquithujene | 1395 | 1391 | 0.21 | 0.14 | 0.14 | 0.13 | 0.12 | 0.12 | 0.17 | 0.25 | 0.03 | 0.15 | 0.16 | 0.16 | 0.16 | 0.12 |
| 6 | β-Elemene | 1399 | 1390 | 1.85 | 1.93 | 1.43 | 1.94 | 1.92 | 0.87 | 1.95 | 1.20 | 0.47 | 1.55 | 1.24 | 1.23 | 2.23 | 1.80 |
| 7 | Cyperene | 1409 | 1398 | 0.70 | 0.80 | 0.64 | 0.80 | 0.80 | 0.31 | 0.60 | 0.59 | 0.16 | 0.62 | 0.62 | 0.64 | 0.73 | 0.72 |
| 8 | α-Cedrene | 1419 | 1411 | 0.20 | 0.20 | 0.19 | 0.20 | 0.15 | - | 0.15 | 0.20 | 0.11 | 0.21 | 0.17 | 0.14 | 0.21 | 0.18 |
| 9 | β-Caryophyllene | 1427 | 1419 | 38.58 | 45.68 | 42.77 | 45.77 | 45.76 | 43.46 | 39.56 | 35.49 | 67.71 | 44.93 | 41.29 | 39.35 | 45.85 | 41.42 |
| 10 | cis-β-Copaene | 1433 | 1432 | 0.31 | 0.23 | 0.23 | 0.22 | 0.22 | 0.12 | 0.21 | 0.46 | 0.21 | 0.20 | 0.74 | 0.94 | 0.17 | 0.16 |
| 11 | γ-Elemene | 1435 | 1436 | 1.46 | 1.82 | 1.79 | 1.85 | 1.86 | - | 1.68 | 1.92 | 0.56 | 1.58 | 1.68 | 1.65 | 1.36 | 1.21 |
| 12 | trans-α-Bergamotene | 1437 | 1434 | 7.87 | 7.55 | 8.31 | 7.57 | 7.58 | 6.57 | 6.88 | 7.53 | 3.46 | 8.32 | 6.70 | 4.75 | 6.96 | 6.32 |
| 13 | Aromandendrene | 1443 | 1441 | 0.44 | 0.37 | 0.42 | 0.37 | 0.38 | 0.43 | 0.36 | 0.48 | 0.12 | 0.38 | 0.66 | 0.57 | 0.34 | 0.33 |
| 14 | trans-β-Farnesene | 1454 | 1456 | 0.43 | 0.33 | 0.32 | 0.31 | 0.30 | 0.22 | 0.29 | 0.41 | 0.06 | 0.36 | - | - | 0.30 | 0.30 |
| 15 | α-Humulene | 1458 | 1454 | 6.26 | 7.24 | 7.56 | 7.20 | 7.23 | 7.79 | 7.38 | 7.74 | 10.57 | 7.87 | 8.50 | 7.83 | 6.07 | 6.86 |
| 16 | allo-Aromadendrene | 1465 | 1460 | 0.50 | 0.47 | 0.50 | 0.46 | 0.46 | 0.39 | 0.41 | 0.70 | 0.20 | 0.43 | 0.51 | 0.71 | 0.41 | 0.40 |
| 17 | Cadina-1(6),4-diene | 1477 | 1463 | 0.65 | 0.57 | 0.50 | 0.56 | 0.56 | 0.34 | 0.48 | 1.10 | 0.28 | 0.51 | 0.73 | 0.66 | 0.53 | 0.56 |
| 18 | γ-Muurolene | 1480 | 1479 | 3.02 | 2.98 | 3.46 | 2.97 | 2.99 | 3.74 | 3.08 | 3.85 | 1.06 | 3.07 | 3.78 | 4.15 | 2.35 | 2.89 |
| 19 | α-Amorphene | 1484 | 1484 | 0.34 | 0.26 | 0.33 | 0.25 | 0.25 | - | 0.29 | 0.37 | - | - | - | - | - | - |
| 20 | Germacrene D | 1486 | 1481 | 1.34 | 1.66 | 2.73 | 1.74 | 1.71 | 2.00 | 2.46 | - | - | 1.85 | - | 0.08 | 2.42 | 2.08 |
| 21 | 9-epi-β-Caryophyllene | 1488 | 1466 | 0.62 | 0.56 | 0.69 | 0.55 | 0.54 | 0.55 | 0.53 | 0.60 | - | 0.66 | 0.41 | 0.29 | 0.35 | 0.49 |
| 22 | β-Eudesmene | 1492 | 1490 | 0.65 | 0.57 | 0.47 | 0.57 | 0.57 | 0.38 | 0.44 | 0.55 | 0.32 | 0.58 | 0.40 | 0.26 | 0.56 | 0.41 |
| 23 | Viridiflorene | 1499 | 1496 | 1.58 | 1.49 | 1.64 | 1.50 | 1.49 | 1.96 | 1.57 | 1.63 | | 1.51 | 1.41 | - | 1.46 | 1.58 |
| 24 | α-Selinene | 1501 | 1498 | 0.61 | 0.59 | 0.52 | 0.57 | 0.57 | 0.55 | 0.46 | 0.61 | 0.31 | 0.59 | 0.54 | 0.40 | 0.54 | 0.45 |
| 25 | Bicyclogermacrene | 1504 | 1500 | 0.10 | 0.11 | 0.22 | 0.14 | 0.13 | 0.15 | 0.22 | - | - | 0.14 | - | - | 0.16 | 0.18 |
| 26 | α-Muurolene | 1506 | 1500 | 1.40 | 0.98 | 1.18 | 0.98 | 0.99 | 0.75 | 1.01 | 2.18 | 0.19 | - | 2.49 | 2.54 | 0.68 | 0.93 |
| 27 | β-Bisabolene | 1513 | 1505 | 3.78 | 2.78 | 3.42 | 2.84 | 2.85 | 2.26 | 2.86 | 5.38 | 1.59 | 2.64 | 3.38 | 4.33 | 1.73 | 2.69 |
| 28 | β-Curcumene | 1516 | 1515 | 0.17 | 0.10 | 0.18 | 0.14 | 0.14 | 0.19 | 0.12 | 0.27 | 0.12 | 0.17 | 0.17 | 0.10 | 0.10 | 0.13 |
| 29 | γ-Cadinene | 1521 | 1513 | 1.43 | 0.95 | 1.57 | 1.20 | 1.21 | 2.09 | 1.36 | 1.94 | 0.66 | 1.35 | 1.71 | 1.90 | 0.97 | 1.33 |
| 30 | δ-Cadinene | 1534 | 1523 | 5.61 | 4.41 | 5.65 | 4.62 | 4.64 | 6.15 | 0.17 | 0.24 | 2.46 | 4.88 | 6.54 | 6.82 | 3.94 | 5.02 |
| 31 | trans-Cadina-1,4-diene | 1541 | 1534 | 0.19 | 0.11 | 0.15 | 0.12 | 0.13 | 0.19 | 0.14 | 0.36 | 0.07 | 0.13 | 0.25 | 0.20 | 0.11 | 0.14 |
| 32 | α-Cadinene | 1546 | 1538 | 0.33 | 0.33 | 0.42 | 0.33 | 0.32 | 0.52 | 0.30 | 0.41 | 0.16 | 0.32 | 0.37 | 0.30 | 0.27 | 0.33 |
| 33 | trans-γ-Bisabolene | 1550 | 1531 | 0.33 | 0.21 | 0.27 | 0.21 | 0.20 | 0.17 | 0.22 | 0.49 | 0.12 | 0.21 | 0.27 | 0.27 | 0.12 | 0.22 |
| 34 | Selina-3,7(11)-diene | 1551 | 1564 | - | 0.13 | 0.17 | 0.13 | 0.12 | 0.12 | 0.16 | - | - | - | 0.29 | - | 0.11 | 0.17 |
| 35 | Caryophyllenyl alcohol | 1578 | 1572 | 0.14 | 0.14 | 0.19 | 0.14 | 0.14 | 0.26 | 0.18 | 0.20 | 0.11 | 0.20 | 0.24 | 0.08 | 0.09 | 0.19 |
| 36 | Caryophyllene oxide | 1590 | 1583 | 0.22 | 0.23 | 0.18 | 0.21 | 0.23 | 0.27 | 0.54 | 0.24 | 0.31 | 0.27 | 0.94 | 0.89 | 0.13 | 0.37 |
| 37 | Gleenol | 1619 | 1587 | 0.22 | 0.17 | 0.13 | 0.16 | 0.17 | 0.11 | 0.22 | 0.23 | 0.09 | 0.15 | 0.23 | 0.25 | - | 0.23 |
| 38 | Junenol | 1627 | 1619 | 0.31 | 0.13 | 0.25 | 0.10 | 0.14 | 0.62 | 0.26 | 0.55 | 0.10 | 0.15 | 0.55 | 0.27 | 0.09 | 0.25 |
| 39 | τ-MuuroloI | 1652 | 1642 | 0.16 | 0.06 | 0.12 | 0.06 | 0.07 | 0.49 | 0.12 | 0.28 | 0.03 | 0.09 | 0.34 | 0.13 | 0.06 | - |
| 40 | δ-Cadinol | 1657 | 1646 | 0.12 | 0.04 | 0.09 | 0.05 | 0.05 | 0.50 | 0.10 | 0.20 | 0.09 | 0.07 | 0.26 | 0.08 | 0.03 | 0.09 |
| 41 | α-Cadinol | 1686 | 1654 | 0.23 | 0.19 | 0.13 | 0.16 | 0.16 | 0.10 | 0.19 | 0.24 | 0.07 | 0.15 | 0.20 | 0.24 | 0.17 | 0.23 |
| 42 | Eudesm-7(11)-en-4-ol | 1691 | 1700 | 0.14 | 0.24 | 0.25 | 0.21 | 0.21 | 0.60 | 0.17 | 0.13 | 0.10 | 0.20 | 0.07 | 0.09 | 0.16 | 0.19 |
| 43 | 16-Kaurene | 2035 | 2043 | - | - | - | - | - | 0.22 | - | - | - | - | - | - | - | - |
| 44 | Manool | 2038 | 2057 | - | - | - | - | - | 0.14 | - | - | - | - | - | - | - | - |
| 45 | Kolavelool | 2043 | - | - | - | - | - | - | 1.34 | - | - | - | - | - | - | - | - |
| 46 | Kolavenol | 2323 | 2297 | - | - | - | - | - | 1.07 | - | - | - | - | - | - | - | - |
| 47 | Methyl kolavenate | 2395 | - | - | - | - | - | - | 0.06 | - | - | - | - | - | - | - | - |
| | | | **Total** | 97.37 | 97.90 | 98.16 | 98.50 | 98.43 | 94.48 | 92.97 | 90.04 | 95.91 | 96.19 | 96.37 | 95.07 | 96.90 | 95.28 |

**Table S1** Continue…

| No. | Compound* | RRI$^{Cal}$ | RRI$^{Lit}$ | 629 | 630 | 863 | 865 | 871 | 873 | 878 | 880 | 888 | 889 | 890 | 891 | 892 |
|---|---|---|---|---|---|---|---|---|---|---|---|---|---|---|---|---|
| | | | | *Unknown Samples* | | | | | | | | *Unknown Samples with Known Location* | | | | |
| 1 | δ-EIemene | 1342 | 1338 | 1.43 | 0.86 | 0.42 | 0.86 | 1.28 | 0.77 | 0.88 | - | 0.31 | 0.30 | 0.31 | 0.06 | 0.05 |
| 2 | α-Cubebene | 1355 | 1351 | 0.57 | 0.09 | 0.43 | 1.25 | 1.71 | 0.76 | 0.92 | - | 0.26 | 0.33 | 0.48 | 0.20 | 0.24 |
| 3 | α-Ylangene | 1379 | 1375 | 0.24 | 0.51 | 0.11 | 0.15 | 0.20 | 0.16 | 0.17 | 0.29 | 0.06 | 0.06 | 0.05 | 0.04 | 0.04 |
| 4 | α-Copaene | 1384 | 1376 | 9.90 | 7.45 | 6.14 | 7.24 | 8.57 | 7.51 | 8.87 | 11.20 | 5.96 | 5.72 | 5.93 | 5.32 | 5.16 |
| 5 | 7-epi-Sesquithujene | 1395 | 1391 | 0.22 | 0.19 | 0.07 | 0.26 | 0.26 | 0.31 | 0.17 | 1.95 | 0.23 | 0.24 | 0.24 | 0.24 | 0.26 |
| 6 | β-Elemene | 1399 | 1390 | 1.74 | 1.29 | 0.98 | 1.48 | 1.70 | 1.49 | 1.73 | - | 1.36 | 1.33 | 1.41 | 1.13 | 1.14 |
| 7 | Cyperene | 1409 | 1398 | 0.73 | 0.64 | 0.51 | 0.65 | 0.62 | 0.49 | 0.62 | 0.72 | 0.38 | 0.38 | 0.40 | 0.26 | 0.25 |
| 8 | α-Cedrene | 1419 | 1411 | 0.21 | 0.17 | 0.09 | 0.20 | 0.18 | - | 0.21 | 0.21 | - | 0.22 | 0.23 | 0.31 | 0.32 |
| 9 | β-Caryophyllene | 1427 | 1419 | 49.92 | 40.96 | 40.66 | 42.23 | 41.23 | 40.91 | 38.83 | 39.07 | 38.50 | 38.25 | 37.20 | 12.57 | 11.61 |
| 10 | cis-β-Copaene | 1433 | 1432 | 0.57 | 0.67 | 0.14 | 0.22 | 0.19 | 0.21 | 0.23 | 0.31 | 0.07 | 0.09 | 0.08 | 0.04 | 0.05 |
| 11 | γ-Elemene | 1435 | 1436 | 1.74 | 1.53 | - | 1.70 | 1.81 | 2.18 | 2.00 | 1.54 | - | - | - | - | - |
| 12 | trans-α-Bergamotene | 1437 | 1434 | 7.84 | 6.20 | 4.49 | 8.28 | 7.22 | 8.94 | 8.70 | 7.67 | 9.68 | 11.61 | 11.22 | 14.24 | 13.75 |
| 13 | Aromandendrene | 1443 | 1441 | 0.36 | 0.67 | 0.45 | 0.48 | 0.42 | 0.27 | 0.46 | 0.49 | 0.27 | 0.32 | 0.37 | 0.34 | 0.32 |
| 14 | trans-β-Farnesene | 1454 | 1442 | 0.23 | 0.19 | 0.19 | 0.21 | 0.18 | 0.13 | 0.46 | 0.27 | 0.44 | 0.45 | 0.48 | 0.59 | 0.60 |
| 15 | α-Humulene | 1458 | 1454 | 7.73 | 8.90 | 7.73 | 7.47 | 7.25 | 7.76 | 6.69 | 6.23 | 7.09 | 6.84 | 7.06 | 1.94 | 1.91 |
| 16 | allo-Aromadendrene | 1465 | 1460 | 0.45 | 0.57 | 0.53 | 0.48 | 0.49 | 0.39 | 0.57 | 0.50 | 0.39 | 0.38 | 0.38 | 0.45 | 0.46 |
| 17 | Cadina-1(6),4-diene | 1477 | 1463 | 0.69 | 0.81 | 0.78 | 0.32 | 0.25 | 0.18 | 0.52 | 0.30 | 0.47 | 0.42 | 0.39 | 0.21 | 0.18 |
| 18 | γ-Muurolene | 1480 | 1479 | 2.31 | 3.92 | 3.72 | 4.06 | 3.81 | 2.97 | 3.31 | 3.21 | 2.85 | 2.75 | 2.89 | 1.85 | 1.81 |
| 19 | α-Amorphene | 1484 | 1482 | - | 0.47 | - | - | - | - | 0.32 | - | 0.32 | 0.30 | 0.32 | - | - |
| 20 | Germacrene D | 1486 | 1481 | 0.22 | - | 0.54 | 0.95 | 1.09 | - | 2.38 | 0.77 | - | - | 0.12 | - | - |
| 21 | 9-epi-β-Caryophyllene | 1488 | 1466 | 0.36 | 0.51 | 0.29 | 0.72 | 0.64 | 0.64 | 0.76 | 0.64 | 1.14 | 1.09 | 1.20 | 2.74 | - |
| 22 | β-Eudesmene | 1492 | 1490 | 0.27 | 0.45 | 0.42 | 0.47 | 0.52 | 0.35 | 0.97 | 0.66 | 1.67 | 1.59 | 1.68 | 2.36 | 2.28 |
| 23 | Viridiflorene | 1500 | 1500 | 1.20 | 1.42 | 2.01 | - | - | - | 1.62 | - | 0.56 | 0.54 | - | - | - |
| 24 | α-Selinene | 1501 | 1498 | 0.35 | 0.51 | 0.61 | 0.59 | 0.60 | 0.44 | 1.02 | 0.64 | 1.39 | 1.32 | 1.42 | 2.15 | 2.06 |
| 25 | Bicyclogermacrene | 1504 | 1500 | - | - | 0.08 | - | - | - | - | - | - | - | - | - | 0.06 |
| 26 | α-Muurolene | 1506 | 1500 | 1.17 | 2.45 | 1.00 | 1.31 | 1.32 | 1.15 | 1.30 | 1.47 | 1.15 | 1.09 | 1.14 | | |
| 27 | β-Bisabolene | 1513 | 1505 | 1.80 | 3.60 | 2.84 | 3.41 | 3.51 | 3.19 | 4.64 | 3.79 | 7.33 | 6.96 | 7.25 | 8.43 | 10.10 |
| 28 | β-Curcumene | 1516 | 1515 | 0.12 | 0.19 | 0.08 | 0.16 | 0.14 | 0.24 | 0.18 | 0.16 | | 0.19 | 0.31 | 0.75 | 0.89 |
| 29 | γ-Cadinene | 1521 | 1513 | 0.81 | 1.80 | 1.85 | 1.82 | 1.84 | 1.75 | 1.49 | 1.53 | 1.57 | 1.46 | 1.53 | 4.25 | 4.88 |
| 30 | δ-Cadinene | 1534 | 1523 | 3.57 | 6.91 | 6.08 | 5.89 | 5.87 | 5.53 | 5.57 | 5.32 | 5.22 | 4.95 | 5.02 | 7.28 | 7.13 |
| 31 | trans-Cadina-1,4-diene | 1541 | 1534 | 0.11 | 0.28 | 0.11 | 0.19 | 0.19 | 0.19 | 0.16 | 0.22 | 0.16 | 0.16 | 0.16 | 0.16 | 0.16 |
| 32 | α-Cadinene | 1546 | 1538 | 0.17 | 0.39 | 0.59 | 0.50 | 0.49 | 0.40 | 0.37 | 0.34 | - | - | - | 0.17 | 0.14 |
| 33 | trans-γ-Bisabolene | 1550 | 1531 | 0.10 | 0.29 | 0.21 | 0.29 | 0.31 | 0.22 | 0.40 | 0.34 | 0.80 | 0.78 | 0.81 | 1.73 | 1.42 |
| 34 | Selina-3,7(11)-diene | 1551 | 1564 | - | - | 0.35 | 0.19 | 0.25 | 0.34 | 0.17 | | 0.41 | 0.36 | 0.30 | 0.50 | 0.37 |
| 35 | Caryophyllenyl alcohol | 1578 | 1572 | 0.10 | 0.24 | 0.58 | 0.21 | 0.20 | 0.36 | 0.15 | 0.13 | 0.26 | 0.25 | 0.26 | 0.12 | 0.16 |
| 36 | Caryophyllene oxide | 1590 | 1583 | | 0.51 | 1.97 | 0.26 | 0.73 | 3.53 | 0.18 | 0.23 | 0.37 | 0.35 | 0.36 | 0.18 | 0.16 |
| 37 | Gleenol | 1619 | 1587 | 0.22 | 0.20 | 0.10 | 0.20 | 0.25 | 0.35 | 0.15 | 0.26 | 0.31 | 0.28 | 0.22 | 0.34 | 0.34 |
| 38 | Junenol | 1627 | 1619 | 0.10 | 0.55 | 0.99 | 0.26 | 0.35 | 0.36 | 0.24 | 0.34 | 0.41 | 0.37 | 0.38 | 0.70 | 0.74 |
| 39 | τ-MuuroloI | 1652 | 1642 | 0.06 | 0.35 | 0.73 | 0.14 | - | 0.20 | 0.12 | 0.17 | 0.34 | 0.35 | 0.34 | 0.11 | 0.13 |
| 40 | δ-Cadinol | 1657 | 1646 | 0.04 | - | 0.66 | 0.09 | 0.13 | 0.20 | 0.09 | 0.12 | 0.58 | 0.55 | 0.57 | 0.12 | 0.14 |
| 41 | α-Cadinol | 1686 | 1654 | 0.23 | 0.18 | 0.10 | 0.14 | 0.17 | 0.25 | 0.15 | 0.19 | 0.21 | 0.19 | 0.16 | 0.18 | 0.19 |
| 42 | Eudesm-7(11)-en-4-ol | 1691 | 1685 | 0.10 | 0.08 | 0.50 | 0.37 | 0.34 | 0.32 | 0.21 | 0.19 | 0.48 | 0.41 | 0.45 | - | - |
| 43 | 16-Kaurene | 2035 | 2043 | - | - | - | - | - | - | - | - | 0.09 | 0.07 | 0.09 | 0.40 | 0.40 |
| 44 | Manool | 2038 | 2057 | - | - | 0.33 | - | - | - | - | - | 0.11 | 0.08 | 0.11 | 0.07 | 0.05 |
| 45 | Kolavelool | 2043 | - | - | - | 1.05 | - | - | - | - | - | 0.11 | 0.08 | 0.11 | 0.02 | 0.03 |
| 46 | Kolavenol | 2323 | 2297 | - | - | 0.52 | - | - | - | - | - | - | - | - | 0.34 | 0.33 |
| 47 | Methyl kolavenate | 2395 | - | - | - | 0.52 | - | - | - | - | - | 0.04 | 0.07 | 0.10 | 0.06 | 0.05 |
| | | | **Total** | 97.98 | 97.00 | 92.55 | 95.70 | 96.31 | 95.44 | 97.98 | 91.47 | 93.35 | 93.53 | 93.53 | 72.95 | 70.36 |

**Table S1** Continue…

| No. | Compound* | $RRI^{Cal}$ | $RRI^{Lit}$ | 893 | 894 | 895 | 896 | 897 | 898 | 899 | 619 | 620 | 628 | 862 | 874 | 877 |
|---|---|---|---|---|---|---|---|---|---|---|---|---|---|---|---|---|
| | | | | *Unknown Samples with Known Location* | | | | | | | *Sample from Commercial Source* | | | | | |
| 1 | δ-EIemene | 1342 | 1338 | 0.06 | 0.18 | 0.17 | 0.18 | 0.72 | 0.96 | 0.89 | 0.05 | - | 0.07 | 0.07 | - | 0.06 |
| 2 | α-Cubebene | 1355 | 1351 | 0.23 | 0.59 | 0.54 | 0.54 | 0.25 | 0.26 | 0.29 | 0.03 | - | 4.65 | - | - | 0.02 |
| 3 | α-Ylangene | 1379 | 1375 | 0.04 | 0.09 | 0.08 | 0.09 | 0.13 | 0.16 | 0.13 | 0.15 | - | 0.01 | 0.15 | - | 0.18 |
| 4 | α-Copaene | 1384 | 1376 | 5.13 | 5.35 | 5.28 | 5.29 | 3.55 | 3.86 | 3.56 | 0.57 | - | 32.93 | 1.27 | 0.28 | 0.63 |
| 5 | 7-epi-Sesquithujene | 1395 | 1391 | 0.25 | 0.21 | 0.22 | 0.22 | 0.08 | 0.09 | 0.07 | 0.16 | - | 0.01 | 0.08 | - | 0.08 |
| 6 | β-Elemene | 1399 | 1390 | 1.13 | 1.15 | 1.10 | 1.14 | 1.53 | 1.64 | 1.52 | 0.61 | - | 6.20 | 1.06 | - | 0.65 |
| 7 | Cyperene | 1409 | 1398 | 0.26 | 0.45 | 0.45 | 0.45 | 0.44 | 0.49 | 0.45 | - | - | 0.02 | - | - | - |
| 8 | α-Cedrene | 1419 | 1411 | 0.31 | - | - | 0.22 | 0.08 | 0.08 | 0.07 | 0.03 | - | - | - | - | - |
| 9 | β-Caryophyllene | 1427 | 1419 | 12.10 | 40.26 | 40.23 | 39.99 | 54.77 | 56.87 | 53.74 | 39.06 | - | 9.07 | 33.66 | - | 43.40 |
| 10 | cis-β-Copaene | 1433 | 1432 | 0.05 | 0.15 | 0.14 | 0.14 | 0.09 | 0.09 | 0.08 | 0.02 | - | 0.03 | 0.05 | - | 0.05 |
| 11 | γ-Elemene | 1435 | 1436 | - | - | - | - | 0.96 | 0.61 | 0.95 | | - | - | - | - | - |
| 12 | trans-α-Bergamotene | 1437 | 1434 | 13.65 | 9.73 | 9.64 | 9.61 | 2.66 | 2.29 | 2.66 | 5.52 | - | 0.08 | 5.18 | - | 6.09 |
| 13 | Aromandendrene | 1443 | 1441 | 0.32 | 0.54 | 0.52 | 0.40 | 0.15 | 0.13 | 0.16 | | - | 0.08 | 0.03 | - | 0.06 |
| 14 | trans-β-Farnesene | 1454 | 1442 | 0.60 | 0.34 | 0.33 | 0.30 | 0.05 | 0.04 | 0.05 | 0.18 | - | 0.06 | 0.13 | - | 0.20 |
| 15 | α-Humulene | 1458 | 1454 | 1.91 | 7.26 | 7.20 | 7.10 | 10.37 | 8.93 | 10.38 | 7.52 | - | 1.08 | 5.00 | - | 7.84 |
| 16 | allo-Aromadendrene | 1465 | 1460 | 0.45 | 0.42 | 0.38 | 0.40 | 0.23 | 0.20 | - | - | - | 2.95 | - | - | - |
| 17 | Cadina-1(6),4-diene | 1477 | 1463 | 0.19 | 0.35 | 0.36 | 0.35 | 0.21 | 0.18 | 0.22 | 0.05 | - | 0.19 | - | - | 0.03 |
| 18 | γ-Muurolene | 1480 | 1479 | 1.80 | 3.96 | 4.01 | 3.94 | 2.95 | 2.48 | 2.91 | 0.24 | - | 0.63 | 0.29 | - | 0.25 |
| 19 | α-Amorphene | 1484 | 1482 | - | - | - | - | - | - | - | 0.10 | - | 0.04 | - | - | - |
| 20 | Germacrene D | 1486 | 1481 | - | 0.72 | 0.24 | 0.44 | 0.05 | - | 0.06 | | - | 1.96 | - | - | - |
| 21 | 9-epi-β-Caryophyllene | 1488 | 1466 | - | 0.89 | 0.88 | 0.90 | 0.17 | 0.13 | 0.15 | 0.38 | - | 0.15 | 0.69 | - | 0.37 |
| 22 | β-Eudesmene | 1492 | 1490 | 2.26 | 0.74 | 0.74 | 0.75 | 0.48 | 0.41 | 0.46 | 0.71 | - | 11.04 | 1.96 | 0.05 | 0.74 |
| 23 | Viridiflorene | 1500 | 1500 | - | - | - | - | - | - | - | 0.19 | - | | | - | |
| 24 | α-Selinene | 1501 | 1498 | 2.07 | 0.91 | 0.91 | 0.93 | 0.41 | 0.35 | 0.41 | 0.45 | - | 6.19 | 1.26 | - | 0.46 |
| 25 | Bicyclogermacrene | 1504 | 1500 | - | - | - | - | - | - | - | 0.28 | - | | 0.43 | - | 0.25 |
| 26 | α-Muurolene | 1506 | 1500 | 1.90 | 1.15 | 1.12 | 1.14 | 0.60 | 0.52 | 0.59 | 0.29 | - | 0.44 | | - | 0.22 |
| 27 | β-Bisabolene | 1513 | 1505 | 10.11 | 4.51 | 4.50 | 4.49 | 0.72 | 0.63 | 0.72 | 8.30 | - | | 9.31 | - | 7.12 |
| 28 | β-Curcumene | 1516 | 1515 | 0.91 | 0.25 | 0.26 | 0.26 | 0.06 | 0.06 | 0.07 | 0.35 | - | | 0.51 | - | 0.32 |
| 29 | γ-Cadinene | 1521 | 1513 | 4.85 | 2.16 | 2.24 | 2.22 | 1.63 | 1.44 | 1.63 | 7.24 | - | 0.73 | 6.91 | - | 6.98 |
| 30 | δ-Cadinene | 1534 | 1523 | 7.10 | 6.30 | 6.49 | 6.39 | 4.52 | 3.99 | 4.50 | 1.09 | - | 8.10 | 1.65 | - | 1.26 |
| 31 | trans-Cadina-1,4-diene | 1541 | 1534 | 0.16 | 0.19 | 0.20 | 0.20 | 0.10 | 0.09 | 0.10 | | - | 0.31 | 0.02 | - | 0.01 |
| 32 | α-Cadinene | 1546 | 1538 | - | 0.62 | 0.65 | 0.65 | 0.69 | - | - | 0.68 | - | 0.02 | | - | |
| 33 | trans-γ-Bisabolene | 1550 | 1531 | 1.71 | 0.47 | 0.45 | 0.48 | - | - | -- | 0.21 | - | | 0.28 | - | 0.43 |
| 34 | Selina-3,7(11)-diene | 1551 | 1564 | 0.53 | 0.29 | 0.32 | 0.31 | 0.94 | 1.11 | 0.91 | | - | | 0.10 | - | |
| 35 | Caryophyllenyl alcohol | 1578 | 1572 | 0.14 | 0.33 | 0.41 | 0.42 | 0.75 | 0.81 | 0.75 | 0.11 | - | 0.03 | 0.05 | - | 0.11 |
| 36 | Caryophyllene oxide | 1590 | 1583 | 0.18 | 0.25 | 0.28 | 0.29 | 0.34 | - | - | 1.03 | - | 0.62 | 0.72 | - | 1.45 |
| 37 | Gleenol | 1619 | 1587 | 0.31 | 0.17 | 0.19 | 0.19 | 0.17 | - | 0.16 | 0.02 | - | 0.11 | | - | |
| 38 | Junenol | 1627 | 1619 | 0.69 | 0.41 | 0.40 | 0.42 | 0.49 | 0.53 | 0.47 | 1.11 | - | 0.01 | | - | 0.19 |
| 39 | τ-MuuroloI | 1652 | 1642 | 0.11 | 0.79 | 0.77 | 0.78 | 0.45 | 0.54 | 0.47 | - | - | 0.40 | 0.03 | - | 0.02 |
| 40 | δ-Cadinol | 1657 | 1646 | 0.13 | 0.72 | 0.69 | 0.73 | 0.57 | 0.66 | 0.58 | 0.01 | - | 0.07 | | - | 0.01 |
| 41 | α-Cadinol | 1686 | 1654 | 0.17 | 0.11 | 0.13 | 0.12 | 0.10 | 0.16 | 0.11 | - | - | | 0.03 | - | 0.01 |
| 42 | Eudesm-7(11)-en-4-ol | 1691 | 1685 | - | 0.67 | 0.70 | 0.70 | 0.56 | 0.64 | 0.57 | - | - | 0.05 | 0.06 | - | 0.06 |
| 43 | 16-Kaurene | 2035 | 2043 | 0.39 | 0.06 | - | 0.07 | 0.10 | 0.11 | 0.11 | - | - | 0.15 | - | - | - |
| 44 | Manool | 2038 | 2057 | 0.06 | - | - | 0.01 | - | 0.01 | - | - | - | - | - | - | - |
| 45 | Kolavelool | 2043 | - | 0.03 | - | - | - | - | - | - | - | - | - | - | - | - |
| 46 | Kolavenol | 2323 | 2297 | - | 0.14 | 0.14 | 0.14 | - | - | 0.14 | - | - | 0.18 | - | - | - |
| 47 | Methyl kolavenate | 2395 | - | 0.03 | - | 0.02 | - | - | 0.02 | - | 2.47 | - | 3.68 | - | - | 1.67 |
| **Total** | | | | 72.32 | 93.88 | 93.38 | 93.39 | 93.12 | 91.57 | 91.09 | 79.21 | 0.00 | 92.34 | 70.98 | 0.33 | 81.22 |

*: Names of compound were provided according to the NIST mass spectral library. The isomer was specified when possible.
$RRI^{Cal}$: relative retention indices calculated against n-alkane.
$RRI^{Lit}$: relative retention indices data from literature.
-: compound not detected or only contain trace amount.
